# Pathological Outcomes in Kidney and Brain in Male Fischer Rats Given Dietary Ochratoxin A, Commencing at One Year of Age

**DOI:** 10.3390/toxins2051100

**Published:** 2010-05-13

**Authors:** Peter G. Mantle, Christopher C. Nolan

**Affiliations:** 1 Centre for Environmental Policy, Imperial College London, London, SW7 2AZ, UK; 2 MRC Applied Neuroscience Group, School of Biomedical Sciences, Queen's Medical Centre, Nottingham, NG7 2UH, UK; Email: c.c.nolan@ntlworld.com

**Keywords:** adenoma, carcinoma, leukemia, renal tumor pathology, latency, neurotoxicity, perfuse-fixed brain histology

## Abstract

Malignant renal carcinoma, manifest in morbid ageing rats, is the striking component of an otherwise silent response after about nine months of exposure to ochratoxin A in the first year of life (daily intake ~100-250 µg/kg body weight). Reasons for the long latency are unclear, as is whether there would be a similar carcinogenic response if toxin exposure started at one year of age. Therefore, 24 male Fischer rats were given 100 µg ochratoxin A as a daily dietary contaminant for 35 weeks from age 50 weeks. Plasma ochratoxin A concentration reached a maximum value of ~8 µg/mL within one month of starting the toxin regimen. No renal carcinomas occurred. Four renal adenomas, two of which were only microscopic, were found among the six rats surviving for 110 weeks. The findings raise new questions about a difference between young adults and mature adults in sensitivity of male rats to the ochratoxin A-induced DNA damage necessary for renal carcinogenesis. A pilot histological study of perfuse-fixed brains of the toxin-treated rats showed no gross abnormalities, correlating with the consistent absence of behavioral or neurological disorders from chronic ochratoxin A exposure regimens in the range 100-250 µg/kg/day during the second half of life. Reasoned questioning concerning ochratoxin A as a neurotoxic mycotoxin is made.

## 1. Introduction

The extensive National Toxicity Program (NTP) toxicological study of ochratoxin A (OTA) in Fischer rats [1] has pointed to renal carcinoma as the notable adverse outcome of lifetime exposure via oral gavage. A similar regimen was employed subsequently, using Lewis and Dark Agouti rats, and confirmed particularly the sensitivity of males [2]. More recently, carcinogenic responses in males given OTA homogenized into feed has established a dose-response relationship for this route of administration of chronic exposure [3,4]. Since the period of OTA exposure necessary to put in place subsequent development of renal cancer was unknown, the necessary period commencing with young adults was measured in Dark Agouti rats [5]. Three months was insufficient and at least six months seemed necessary to be carcinogenic. However, complimentary experiments showed that about nine months exposure would cause carcinoma in about 20-25% of individuals. This implies that OTA exposure during the latter half of a rat’s life may be completely ineffectual for renal carcinogenesis, either because it exceeds what is necessary for causing genetic change or because of physiological changes as mature adults age. Thus an experiment was designed at least to show whether there is an intrinsic difference between the first and second halves of rat life with respect to sensitivity to OTA carcinogenicity. As in other recent studies at Imperial College London, opportunity has been taken to study tumors in some depth, commencing exposure at one year of age, as opposed to just recording incidence. Measurement of outcome was complicated, as in the NTP study, by the mononuclear leukemia typical of Fischer rats, but the findings can be more readily matched to the NTP protocol using that strain. 

There are several reports of toxic effects of OTA on laboratory rodent brain. For example, anatomical differences were seen in cerebral cortex of mice at six weeks of age, born to an animal given a rather large single dose of OTA (3 mg/kg body weight, i.p.) on day 10 of gestation [6]. In rats, comparing a range of doses given by oral gavage for four weeks to young or old females, gross histological change was noted as vacuolation in cerebellar medulla and ventral parts of the brain-stem, increasingly dose-related in the young rats but inversely so in old rats [7]. Authors concluded that brain may be a primary toxicity target organ for OTA and that old rats are more sensitive than young ones. However, no adverse neurological effects were recorded in the NTP study during two years of OTA exposure, and no abnormal rat behavior was evident throughout all recent lifetime OTA rat studies at Imperial College London. Thus, pilot histological study of perfuse-fixed brains was added to the present experiment, necessarily made at or near the stage of natural mortality, which did not significantly alter group survival dynamics while ensuring that brains came from animals in good physical condition, alert and well coordinated.

## 2. Methods

### 2.1. Animal experiment

Twenty-four male F344 (Fischer) rats (Harlan) were maintained on standard maintenance diet (RM1 (14.4% protein) Special Diet Services, Witham, Essex, UK) until one year of age, Thereafter, caged in groups of three, they were fed daily with 20 g each of the same diet, though in powdered form into which had been homogenized an aqueous infused suspension of shredded wheat fermented with a culture of *Aspergillus ochraceus*, exactly as previously described [3,4]. OTA content of the contaminated diet was 5 ppm, requiring dilution of the toxin-rich fermented product [8] by a factor of at least 10^3^. All feed was consumed each day. Animal welfare was monitored daily and any animal that became jaundiced, lost significant weight or became moribund was euthanized. 

Thirty-five weeks after OTA exposure commenced, a period found to be carcinogenic when applied to young rats [3,5,9], nine of the surviving animals ceased OTA exposure while the other 10 continued on the contaminated diet for life. Sixteen weeks later, nine of the surviving rats were maintained overnight (18 hours) in metabolism cages with food and water *ad libitum*, for collection of urine into a vessel containing 1 mL of a 1% aqueous solution of sodium azide.

### 2.2. Urinalysis

Automated urinalysis for creatinine, protein, calcium, sodium, potassium, urate, urea and phosphate was performed in the Chemical Pathology Laboratory at St. Mary’s Hospital, Paddington, London, using an Olympus AU640 instrument with methodology described in [10]. 

### 2.3. OTA in blood plasma

Quantitative measurement was made at the Central Science Laboratory, York by validated methodology as previously described [11]. 

### 2.4. Brain Histopathology

Survival of some animals to 115 weeks of age coincided with the age of the formal NTP end-point for toxicological studies (105 weeks of experiment commencing with rats of about 10 weeks of age). Thus, the opportunity was taken to explore the histological status of brain after a year’s exposure to OTA as mature adults, but while animals yet showed no clinical morbidity. 

**Figure 1:**
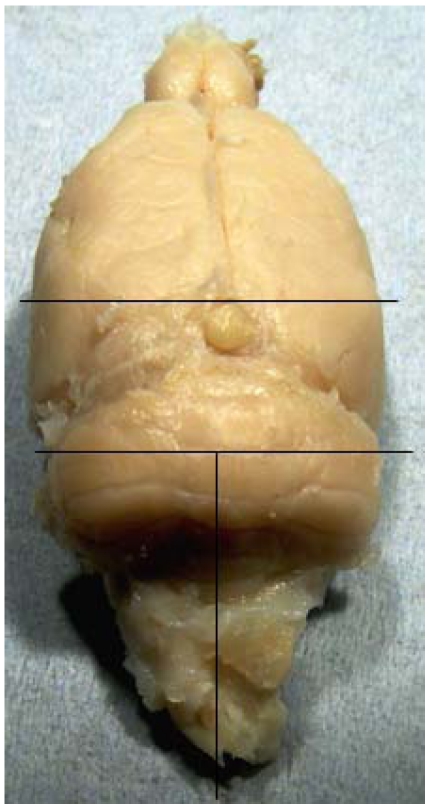
Brain sectioning for histology.

Six rats were anaesthetized with fluothane/oxygen, further sedated with pentobarbitone sodium (100 mg, i.p.), and killed by transcardiac perfusion, following a brief flush of physiological saline, with formalin-acetic acid solution (10%:2%) in distilled water at a pressure of 100-120 mm Hg for 12 min. Heads were submerged in the same fixative for three hours before dissection. Whole brains were stored in 4% formalin at 4 °C. Subsequently, the brains from two rats treated continuously with OTA, and from one which ceased OTA treatment after 35 weeks, were selected for histological study; brains were divided along saggital and coronal aspects (Figure 1), and tissues embedded in wax blocks employing chloroform as the clearing solvent. Sections (10 µm) were stained with hematoxylin and eosin in the Breast Pathology laboratory, Guy’s Hospital, London. 

### 2.5. General histology

Standard wax-embedded blocks were also prepared from kidneys and testes, and sections (3-4 µm) were stained with hematoxylin and eosin also at Guy’s Hospital. 

## 3. Results

**Figure 2:**
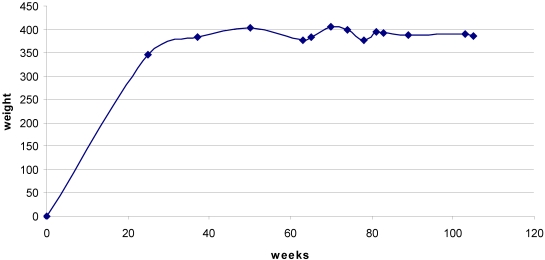
Mean body weight from 25 weeks before OTA treatment commenced until the 105 week stage. Undulations after 50 weeks reflect premature death of some individuals (c.f. Figure 4), but a steady value was generally maintained.

Rats maintained body weight throughout the 35 weeks of exposure to OTA and beyond (Figure 2).

**Figure 3:**
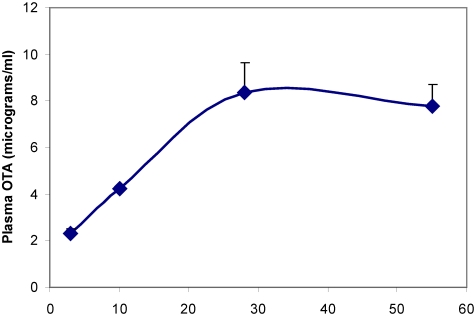
Accumulation of OTA in blood plasma during the first eight weeks on contaminated diet.

Plasma OTA concentration reached a steady state value of ~8 µg/mL within one month of transfer to the contaminated diet (Figure 3). End stage concentration measured in some rats at least 100 weeks old (Table 1) showed lower values attributed to ageing, and elimination to near the limit of detection 16 weeks after ceasing OTA exposure.

**Table 1:** Plasma OTA concentration measured in blood from six rats at necropsy.

Age (weeks)	OTA continuous since 50 weeks old	OTA ceased 16 weeks previous to this blood sample
100	2.0 µg/mL	
101	2.5 µg/mL	1.8 ng/mL, consistent with ~13 plasma half lives elapsed sinceOTA exposure ceased.
109	4.6 µg/mL	
109	1.1 µg /mL	
110	0.3 µg/mL	
110	0.6 µg/mL	

Fifty-six weeks after OTA treatment commenced, difference was found in measured urine parameters, between the group which continued OTA treatment for a further 21 weeks (Table 2) and the group in which OTA treatment had been discontinued, only in increased excretion of protein in some individuals in response to continuing ingestion of OTA.

**Figure 4:**
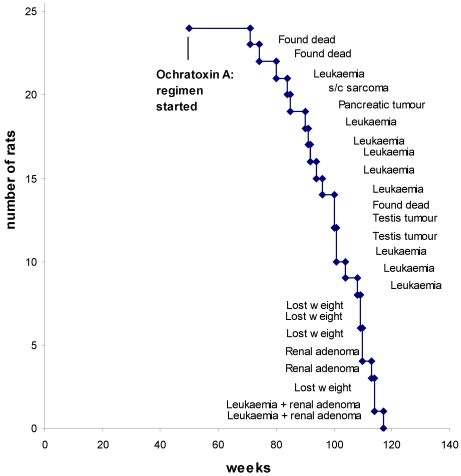
Survival dynamics annotated with principal outcome for each rat.

Survival dynamics from the start of OTA exposure is shown in Figure 4, annotated with a principal feature for each case. Only three of the rats died unexpectedly overnight. The typical mononuclear leukemia, that potentially complicates assessment of renal tumorigenic response in F344 rats by premature need for euthanasia, became evident in nine animals (38%). These were distributed as usual across the last quarter of life. However, careful scrutiny of renal histology meant that the first decedence (at 80 weeks) could have been before potential proliferation of a recognizable renal neoplastic lesion attributable to OTA had occurred. Similarly, euthanasia of the cases of lip sarcoma and pancreatic tumor at 84 and 85 weeks might have precluded evidence of a late-developing renal neoplasm. Later, a testis tumor was found in a rat in each of the groups, whether continuing OTA ingestion or discontinued. 

**Table 2:** Comparative uro-analytical findings from groups of rats surviving at ~2 years of age. They had been consuming ochratoxin A-contaminated diet for 35 weeks from 50 weeks of age. At 85 weeks of age, one group remained on the OTA diet while the other reverted to normal rat diet. Ultimate principal oncological findings are given for each animal. Kidney indicates the individuals in which renal adenoma was later found. Units; urine (18 h, mL), creatinine (mmol/L), protein (g/L), other solutes (mmol/mmol creatinine).

Prospective oncology	Volume	Calcium	Urate	Creatinine	Protein	Sodium	Potassium	Phosphate	Urea
**Continuous OTA**									
Testis	7.6	0.24	0.30	9.75	0.72	3.6	17.53	4.08	76.18
Testis *	11.7	0.53	0.23	6.14	1.84	9.15	22.65	3.32	77.22
Testis *	8.0	0.12	0.25	8.35	1.11	6.08	16.17	4.21	68.14
Kidney	8.1	0.16	0.20	10.77	0.64	6.13	15.69	3.14	67.49
Testis, Leukemia	12.0	0.36	0.45	5.81	2.25	6.80	16.88	6.12	64.41
Mean	9.5	0.28	0.29	7.56	1.31	6.35	17.78	4.17	70.69
**OTA ceased for 22 weeks**									
Kidney (unilateral, total; 6.4 g)	12.4	0.11	0.28	7.84	0.62	5.33	14.54	3.35	56.12
Testis, Leukemia, Kidney	12.0	0.25	0.39	7.73	0.35	6.53	15.39	2.80	61.80
Testis, subcutaneous sarcoma	13.0	0.46	0.28	5.41	0.88	8.17	19.77	4.17	76.11
Leukemia, Kidney *	10.7	0.57	0.27	8.20	0.87	6.14	16.47	3.33	59.54
Mean	12.0	0.35	0.31	7.30	0.68	6.54	16.54	3.41	63.39

* Histology of perfusion-fixed brain studied.

**Figure 5:**
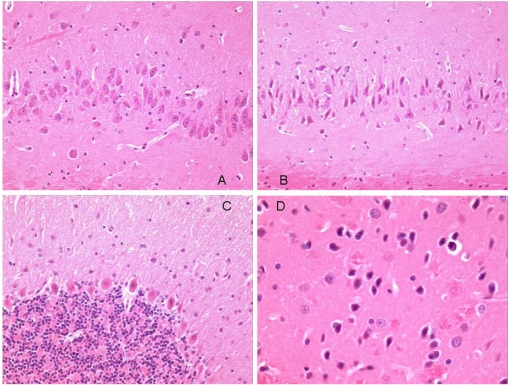
Renal adenomas. A: rat 1, junction between adenoma and kidney cortex. Note the eosinophilic (necrotic) region top left; B: rat 2, tumorous kidney *in situ*; C: rat 2, excised tumorous kidney; D: rat 2, tumorous kidney in section showing renal papilla; E: rat 2, typical adenoma histopathology; F: rat 3, micro-adenoma *in situ* with central eosinophilic (necrotic) region.

Correlated with advanced ageing at 110 weeks or more, a focal unilateral renal adenoma was found in four rats (Figure 4). All were solid compression tumors within the kidney capsule and without any evidence of distant metastasis. The first (rat 1) was small (7 × 8 mm), distorting the kidney, but cellular proliferation internally retaining a tubular arrangement typical of adenoma (Figure 5A). The second (rat 2) was larger (16 × 12 mm, Figure 5B and 5C), preserving the papilla part of the kidney (Figure 5D, total tumorous organ weight 6.4 g) but probably destroying excretory function. Tumor histology was of typical adenoma. The third (rat 3) was only detected microscopically. It was comprised of only two or three adjacent swollen tubules with regular internal cellular proliferation (Figure 5E); a central group of necrotic cells with pyknotic nuclei is consistent with increasing isolation from vasculature as the tumor proliferated in this aged animal. The fourth was in the last surviving rat and was a spherical tumor (3 mm) diameter, located centrally, not notably distorting the kidney, and only detected during histological preparation. It was probably not of current clinical significance.

Histology of the three brains showed similar features. Generally there was evidence of swollen astrocytes, and "haloes" around neurons throughout the brains, which is probably a fixation/processing artifact; this is difficult to avoid even after perfuse-fixation. In both a continuously OTA-treated rat and the one treated for the shorter period, there was pronounced swelling around the lateral and third ventricles which is probably also artifactual, although this was not so prominent in the other continuously treated rat.

**Figure 6:**
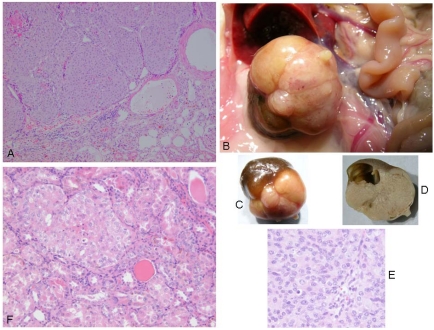
Photomicrographs of regions of perfuse-fixed brain of rat consuming OTA-contaminated diet during weeks 50 to 109 of life. A: hippocampus, normal pyramidal neurons, ×20 objective; B: hippocampus, eosinophilic pyramidal neurons, ×20 objective; C: cerebellum, eosinophilic Purkinje cells, ×20 objective: D: striatum, eosinophilic neurons, ×40 objective.

**Figure 7:**
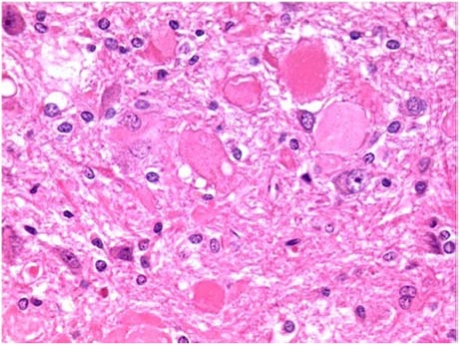
Photomicrograph of cuneate of perfuse-fixed brain of rat consuming OTA-contaminated diet during weeks 50 to 109 of life, illustration of eosinophilic bodies (×40 objective).

Neurons in most regions of all rats appeared fairly normal. However, there were some eosinophilic Purkinje neurons in the cerebellum of all animals (Figure 6C). One of the continuously treated animals also had eosinophilic neurons in the ventral striatum and dorsal hippocampus (Figure 6B and Figure 6B). The somewhat angular appearance of the cells, and the lack of an obvious concomitant increase in cellularity (microglial/astrocytic response) around them, suggests that these appearances are also artifactual. From experience, Purkinje cells are particularly prone to this. More significant, may be the large eosinophilic bodies in the cuneate nucleus (Figure 7), located in the brainstem just below the back of the cerebellum. These were present in all animals, and in one of the continuously treated animals there was some evidence of them in the fiber tract leading up to the nucleus.

## 4. Discussion

Throughout the study, exposure to OTA as a dietary contaminant was well tolerated as indicated by rat body weight, urinalysis and survival, which were not notably different from the control (untreated) groups already described for concurrent lifetime studies [3,4]. Maximum plasma OTA concentration was similar to that achieved in [3] and which caused metastasizing renal carcinomas. Further, the kinetics of plasma OTA accumulation during the first month of OTA exposure followed the pattern predicted from classical pharmacokinetic study also in Fischer male rats [12]. In the present context, where development of several cases of renal carcinoma could reasonably have been expected in a study not shortened for all rats to the standard two years of NTP studies, it is therefore notable that carcinoma did not occur. The findings at least demonstrate the value of long term whole animal experiments in assessing the real toxicological significance of OTA, which is much more difficult to perceive either from acute animal studies or from experiments on tissue-cultured cells.

Unlike in other studies, in which exposure to feed contaminated with OTA commenced in young rats [3,4,5,9] but after which there was a latency period of many months before renal tumors were found, the present protocol allowed much less opportunity for expression of long latency. Nevertheless if, as recently reported [9], about 35 weeks of OTA exposure was sufficient for unilateral renal tumorigenesis to be put in place, the first adenoma here was only found after a further six months of continuous OTA exposure, at an age close to the normal endpoint of NTP toxicological studies. Notably the other three small adenomas all occurred in rats given only the basic 35 weeks of OTA exposure, but found at an age beyond a classical two year endpoint.

The present findings raise questions about the mechanism and kinetics of OTA renal tumorigenesis and its components of promotion, adenoma proliferation and aneuploidization, and transition to infiltrative malignant carcinoma with potential for distant metastasis. At least part of the mechanism seems to involve direct covalent interaction with DNA [13].

It is now nearly half a century since OTA was discovered. Although a potent renal carcinogen in the male rat, its significance for human health remains obscure. It is thus the more urgent to determine whether the rat is a valid experimental model for any human carcinoma.

The eosinophilic bodies in cuneate nuclei of brainstems seem to be real changes, although they do not appear to have stimulated a cellular reaction. They are reminiscent of dystrophic fiber terminals found in the gracile and cuneate tracts/nuclei of three month old mice treated with organophosphorous compounds. Such swellings occurred in control mice, but less frequently than in dosed animals. However, the present observations are likely to be of ageing-related changes in the present very old male rats. Further histopathological study of these brains, and others from the present experiment (all are available on request), might include labeling of sections for GFAP and neurofilaments to aid interpretation of the swellings. It is concluded that none of the minor histological features could reasonably be attributed other than to ageing and/or tissue processing. There was no evidence of adverse neurohistopathological effect of continuing the dietary OTA exposure for a further five months during ageing; histology in Figure 6 is of a non-leukemic rat given OTA for a total of 59 weeks. 

In contrast, a distribution study [14] tracing OTA (0.4 µg) given by injection into a lateral ventricle, none was distributed to cerebellum, striatum or ventral mesencephalon and none was transferred to blood. Also, OTA was given by oral gavage (289 µg/kg/day) for eight days to pre-pubertal male rats. Of the proportion then found in brain (not stated), one-third was in the cerebellum but only 3%-5% was each in the striatum, hippocampus and ventral mesencephalon. Consequently, authors designated these regions as the main OTA targets in ‘adult’ rat brain. Unfortunately, OTA concentration was not measured in the blood after chronic OTA administration, but from the present study it might be estimated at 2-3 µg/mL. Since brain vasculature had not been flushed prior to further analysis, the small amounts of OTA measured in brain structures may be attributed to OTA in brain vasculature. Claims of OTA accumulation in kidney can be subject to similar misinterpretation [13].

Another acute (i.p.) study in mice [15] found DNA damage, DNA repair and oxidative stress across six brain regions, but only at rather high OTA dose. That study was extended to question involvement of low dose exposure in parkinsonism [16]; however, biologically-active dose vastly exceeds that of natural human intake.

Aggregating brain cell culture, as an *in vitro* model for neurotoxicological study, has been applied to OTA for investigating gene expression mechanisms underlying neurotoxicity [17]. The elegant technique allowed intimate contact between aggregates and OTA in a nutrient medium and observed changes related to brain inflammatory response. Such might be significant *in vivo* if OTA has access to developing fetal brain. In contrast, in the present study, OTA circulated bound to serum proteins and there is no compelling evidence of significant transfer out of brain vasculature. Unqualified description of naturally-occurring OTA as a ‘neurotoxin’ may therefore be misleading. 
